# Cognitive Benefits From a Musical Activity in Older Adults

**DOI:** 10.3389/fpsyg.2019.00652

**Published:** 2019-03-28

**Authors:** Veronika Diaz Abrahan, Favio Shifres, Nadia Justel

**Affiliations:** ^1^ Laboratorio Interdisciplinario de Neurociencia Cognitiva (LINC), Centro de Estudios Multidisciplinarios en Sistemas Complejos y Ciencias del Cerebro (CEMSC3), Escuela de Ciencia y Tecnología (ECyT), Universidad Nacional de San Martín (UNSAM), Buenos Aires, Argentina; ^2^ Consejo Nacional de Investigaciones Científicas y Tecnológicas (CONICET), Buenos Aires, Argentina; ^3^ Universidad Nacional de Córdoba, Córdoba, Argentina; ^4^ Departamento de Música, Facultad de Bellas Artes (FBA), Laboratorio para el Estudio de la Experiencia Musical (LEEM), Universidad Nacional de La Plata (UNLP), La Plata, Argentina

**Keywords:** cognitive reserve, musical strategy, improvisation, memory, aging

## Abstract

The aging population is growing rapidly. Proposing interventions that enhance the cognitive functions or strategies that delay the onset of disabilities associated with age is a topic of capital interest for the biopsychosocial health of our species. In this work, we employed musical improvisation as a focal environmental activity to explore its ability to improve memory in older adults. We present two studies: the first one evaluated neutral memory using the Rey Complex Figure (RCF) and the second one evaluated emotional memory using International Affective Picture System (IAPS). A group of 132 volunteers, between the ages of 60 and 90, participated in this investigation. Fifty-one of them were musicians with more than 5 years of formal musical training. After acquisition of neutral (Study 1) or emotional (Study 2) information, the groups of older adults were exposed to music improvisation (experimental intervention) or music imitation (control intervention) for 3 min. We then evaluated memory through two tasks (free recall and recognition), by means of immediate and deferred measures (after a week). We found a significant improvement in memory among participants involved in music improvisation, who remembered more items of the RCF and images from IAPS than the imitation group, both in the immediate and deferred evaluation. On the other hand, participants who had musical knowledge had a better performance in neutral visual memory than non-musicians. Our results suggest that a focal musical activity can be a useful intervention in older adults to promote an enhancement in memory.

## Introduction

Nowadays, there is an increase in life expectancy, which is highly positive for the human being, although it brings with it a decline in our cognitive functions (Christie et al., 2017). It is estimated that by 2050 there will be 114 million people with dementia, this condition being one of the major causes of disability and dependence in the older adult population (World Health Organization, 2012; Iuliano et al., 2015). For this reason, proposing interventions that enhance the cognitive functions or strategies that delay the onset of disabilities associated with age is a topic of capital interest for the biopsychosocial health of our species (Kramer et al., 2004). For example, treatments that enhance cognitive abilities could be promoted in each life stage, from childhood to old age.

Memory is one of the cognitive skills most affected by aging (Nyberg et al., 2003; Park and Festini, 2017). This function could be defined as the capacity to learn, store, and retrieve information (Tulving, 2002; Squire and Wixted, 2011). There are several memory subsystems; the one mostly affected by aging is episodic memory (Friedman, 2013). At the same time, emotional memory could be considered a part of episodic memory, and it is defined as better storage and recall of the events associated with emotional factors, i.e., those events that have an emotional load are better remembered than the neutral ones (Cahill and McGaugh, 1995; Bermúdez-Rattoni and Prado-Alcalá, 2001). Evidence showed that older adults had a decrease in episodic memory, but emotions could work as enhancers and compensate for this deficit (Moayeri et al., 2010).

Several strategies or environmental interventions, in addition to lifestyles, have been investigated mainly to improve cognitive functions and to prevent and/or delay cognitive deficits. Such interventions include learning other languages (Abutalebi et al., 2015), physical activity (Loprinzi et al., 2018), and music (Schneider et al., 2018). In particular, music makes unique demands on our nervous system (Justel and Diaz Abrahan, 2012), and therefore, over the last years, music and each of its components have been used as a tool to investigate human cognition and its underlying brain mechanisms, because music affects the cortical and subcortical areas (Pantev and Herholz, 2011; Koelsch et al., 2018). Some studies show that listening to music improves cognitive skills such as fluency (Thompson et al., 2006), working memory (Mammarella et al., 2007), and recognition memory (Ferreri et al., 2013), among others. For example, background music was investigated as a focal and acute strategy that could improve cognitive skills. This technique refers to any music that is played while the listener’s primary attention is focused on another task or activity (Bottiroli et al., 2014). Different studies about the effect of background music have shown some improvements on cognitive abilities. For example, Judde and Rickard (2010) performed a study in which participants listening 3 min of music after the acquisition of information and they had a better recognition memory 1 week later. However, there is some evidence of reduced cognitive performance when music is present (Kämpfe et al., 2010; Rickard et al., 2012).

Furthermore, other investigations indicate that musical production could have even more beneficial effects than musical perception (Lappe et al., 2008; Fancourt et al., 2014). There is some research about music production, as a focal intervention, in the field of neurologic music therapy (Thaut et al., 2009; Thaut and Hoemberg, 2014), but none of them focused on the effects of music production on memory. Besides, the studies distinguish how music and its components affect people with and without formal musical knowledge (Zuk et al., 2014; Schlaug, 2015; Zhao et al., 2017). In general, because of their extensive training affecting the anatomical and functional organization of their brains, musicians have been shown to have a greater cognitive reserve than non-musicians (Hanna-Pladdy and Gajewski, 2012), and hence, their memory would be less compromised over the years (Talamini et al., 2018). In addition, the protective effect of playing an instrument is greater than that of other leisure activities (Amer et al., 2013). For example, some studies indicated that music training has shown improvements in the cognitive functions of older musicians compared with non-musicians, such as memory, naming, and executive functions, among others (Hanna-Pladdy and MacKay, 2011).

Among the interventions that involve musical production, musical training is the one that has received the most attention. Training includes learning how to play an instrument, and most studies evaluate the effect of moderate or long-term learning (Barrett et al., 2013), leaving a gap as far as focal interventions are concerned. Another intervention that involves musical production is musical improvisation, which is defined as an example of musically creative behavior, conceived as an original and novel process requiring divergent thinking (Bengtsson et al., 2007; Manzano and Ullén, 2012; Diaz Abrahan and Justel, 2015). Research is scarce in this area, and most studies emphasize the use of improvisation in musicians (Limb and Braun, 2008); assuming that improvising musically implies having some degree of expertise in music. However, it is also used with people without musical training as a technique for the patient population (e.g., neurological music therapy, Thaut et al., 2009). In this perspective, music improvisation is conceived as the combination of sounds created in a specific framework inside an environment of trust, which is established to address the needs of the participant or patient (Wigram, 2004). In this sense, music improvisation is not only performed by musicians, but it is also a real-time ability that every person has (Wigram, 2004). Still, research on the use of the musical improvisation technique in people without a pathology and in non-musicians is infrequent. In addition, older people are unlikely to begin learning an instrument at an advanced age. Therefore, providing the opportunity of a focal intervention where the participants play instruments and create something novel in groups, without long-term demands, could result in low dropout rates.

The main goal of this work was to investigate the effect of a focal environmental activity as a possible memory improvement technique in older adults. We evaluated whether there were differences between neutral and emotional memory and between participants with and without formal musical knowledge. The intervention employed was musical improvisation, because it involves a musically creative behavior that may be implemented in musicians or non-musicians and because this focal/acute technique is used with older adults. We expected musical improvisation to improve memory and musicians to perform better than non-musicians in the memory evaluations. Finally, we hypothesized that information with emotional content would be better remembered than neutral information.

## Study 1

### Materials and Methods

#### Participants

Sixty-nine volunteers (75% female participants) between the ages of 60 and 90 (*M* = 74.16; *SD* = 1.1) participated in this study. Twenty-six were musicians (M) with more than 5 years of formal musical training (schools, institutes, music conservatories). Forty-three were considered non-musicians (NM). An *a priori* power analysis suggested that *N* = 57 would be adequate to provide 0.60 power (software G*power, Faul et al., 2007). They were recruited from different senior cultural centers through online announcements. Participant exclusion criteria included visual or hearing impairment, amusia, or any music-related pathology, cognitive impairment, and depression. Each participant signed a written informed consent form and completed a questionnaire where socio-demographic and musical expertise information was requested. The procedure was approved by the University of Buenos Aires Ethics Committee.

#### Measures

##### General Cognitive State Evaluation

As depressive symptomatology may affect memory, we administered the Yesavage Geriatric Depression Scale (GDS, Sheikh and Yesavage, 1986; Martinez de la Iglesia et al., 2002), which measures depression specifically in older adults by assessing anhedonia, sadness, loss of interest, etc. Scores between 0 and 10 are considered to be within the normal range, scores 11–14 show sensitivity to depression, and scores over 14-signal show depression. The participants with a score of 11 or more were excluded. The Mini Mental State Examination (MMSE, Folstein et al., 1975) was used to rule out cognitive impairment. The MMSE is a screening test that measures dementia symptoms. Scores between 9 and 11 are considered to be within the dementia range, scores between 12 and 24-signal cognitive impairment, and scores between 24 and 26 suggest sensitivity to dementia. For schooled participants under 75 years of age, 27 points was the cut score; when the schooled participants were over 75 years old, 26 was the score selected to exclude participants (Butman et al., 2001). Both, the GDS and MMSE were administered individually.

##### Neutral Memory Evaluation

The material for the neutral memory task was the Rey Complex Figure (RCF; Meyers and Meyers, 1995). It is a figure with 18 different items that compose a larger image.

#### Instrumental Setting

For the musical experiences (imitation or improvisation), participants were allowed to choose percussion instruments (e.g., drums, maracas, bells, wood blocks, shakers, tambourine) or melodic/harmonic instruments (e.g., guitar, melodica, xylophone, flutes). These instruments were included because they were easy to handle.

##### Musical Interventions

###### Music Improvisation (Experimental Condition, EXP)

**Figure 1:**
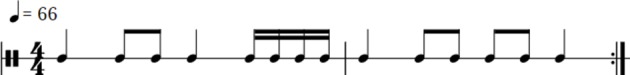
Rhythmic base-pattern presented by the researcher to guide both music reproduction and improvisation that participants were asked to perform with a set of basic instruments.

The first author (a music therapist) performed a rhythmic pattern repeatedly during 3 min as a base for an improvised performance by the participants playing their instruments. This pattern was performed with a percussion instrument at a medium volume (Figure 1; Berkowitz and Ansari, 2008, 2010; Manzano and Ullén, 2012; Pinho et al., 2016). Participants chose any instrument and improvised musical patterns with instruments or their voices or bodies, spontaneously creating some musical feature according to the context provided by the base-pattern. The instructions included playing without restrictions: the researcher proposed a free improvisation based on the same rhythmical pattern used in REP intervention (Figure 1). Such a rhythmical baseline was introduced in order to guide non-musician participants because pilot studies had shown that without such a guidance participants could not follow the improvisation directions.

###### Imitation (Control Condition, CTRL)

The same researcher who conducted the musical improvisation performed the same rhythmic pattern repeatedly during 3 min as a model to be imitated by the participants with their instruments. This pattern was performed in the same percussion instrument at a medium volume. In this intervention, the participants imitated the pattern for 3 min (Gilbertson, 2013). The instructions included imitating the pattern heard as faithfully as possible, avoiding variations or new musical materials. This intervention was meant to control for possible effects of movements, music perception, musical instruments, among others, that could explain the results.

#### Experimental Design

Because there were two interventions (EXP vs. CTRL) and the participants had different musical expertise (M and NM), a 2(Intervention) × 2(Training) experimental design was run, with four groups with the following number of subjects: (1) M/EXP: musicians’ improvisation group (*n* = 15); (2) M/CTRL: musicians’ imitation group (*n* = 11); (3) NM/EXP: non-musicians’ improvisation group (*n* = 22); and (4) NM/CTRL: non-musicians’ imitation group (*n* = 21). Participants were randomly and blindly assigned to the different groups, and they were always tested in groups, with a minimum of four and a maximum of 10 participants, in order to control the involvement of each participant in the music performance.

##### Procedure

The study was divided into two sessions with a one-week intersession interval. The first session consisted of four immediately consecutive phases. In the first phase (information phase, about 15 min), the participants signed the informed consent form and completed the socio-demographic and musical expertise questionnaire. In this step, we also evaluated the general cognitive state with MMSE and GDS. In the second phase (acquisition, 9 min), the participants watched the RCF and they were asked to copy it (they were supplied with pencil and paper).

In the third phase (treatment phase, about 3 min), the participants were exposed to the musical interventions (improvisation or imitation). The following directions were given during the music improvisation intervention: *“We will listen to a rhythmic base, from which you have to create something musical as a group. This rhythmic base will help you to start the improvisation at any time you want. You can use instruments, your voice or your body. It is important to listen not only to the base but also to your own group*.” In the imitation intervention (control condition), the following directions were given: “*We will listen to a rhythmic base and, anytime you want, you can start to imitate me. You can use instruments, your voice or your body.”* Before starting, the researcher corroborated that all the participants understood the instructions. Then, they chose freely the musical instrument that they wanted to play, and they performed the improvisation or imitation task in groups for 3 min.

Soon afterwards, in the fourth phase (test phase, about 11 min), a two-task test was run. Participants were given paper and pencil to drawn from memory the RCF (*Immediate Free Recall* task), and then 12 target items of the RCF were mixed with 12 new items and participants were asked to indicate whether they had seen the item before or not (*Immediate Recognition* task).

**Figure 2:**
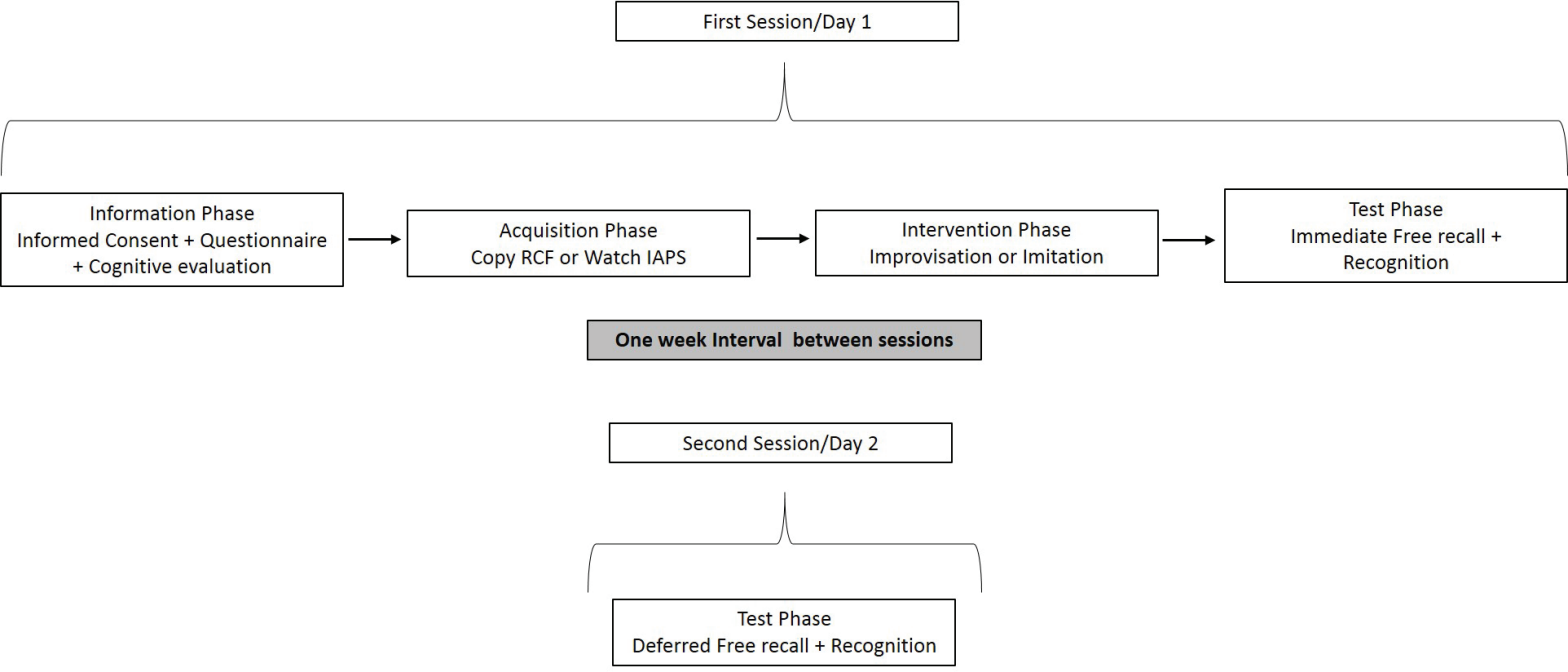
Scheme of the experimental procedure.

The second session (11 min) was held a week later, when the two-task test was run again (*Deferred Free Recall* task and *Deferred Recognition* task; see Figure 2 for a schematic design of the procedure).

##### Data Analysis

Age, years of formal education, and years of musical education were analyzed independently *via* univariate analysis of variance (ANOVA), where *Intervention* (improvisation vs. imitation) and *Training* (musicians vs. non-musicians) were the between-factors.

Copy and free recall (immediate and deferred) of the RCF were evaluated by means of the following procedure: Each of the 18 components of the RCF was evaluated according to whether it was well-drawn and correctly located (2 points), well-drawn but incorrectly located (1 point), badly drawn but correctly located (1 point), badly drawn but recognizable (0.5 points), and badly drawn and incorrectly located (0 points). The maximum final score could amount to 36. Because musicians had more years of education than non-musicians and because there were differences in the copy of the RCF (data shown in Results section), recall and recognition (immediate and deferred) were independently analyzed *via* ANCOVA with *Intervention* (improvisation vs. imitation) and *Training* (musicians vs. non-musicians) as the between-factors and *Education* and *Copy* as the co-variables.

*Post hoc* least-significant difference (LSD) pairwise comparisons were conducted to analyze significant interactions. The partial Eta square ($\eta_{p}^{2}$) was utilized to estimate effect size. The alpha value was set at 0.05, and the SPSS software package was used to compute descriptive and inferential statistics.

## Results

### Socio-Demographic Characteristics and General Cognitive State

The final sample consisted of 64 participants, because five evaluations were discarded due to cognitive deficit and/or depression; the final number of participants per group were as follows: (1) M/IMP = 13; (2) M/REP = 10; (3) NM/IMP = 21; and (4) NM/REP = 19. The general cognitive state values (MMSE and GDS) are depicted in Table 1.

**Table 1:** Socio-demographic and general cognitive state data.

Groups	Age	Education	Musical educ.	MMSE	GDS	Copy
M/EXP	70.93 ± 3.51	15.27 ± 0.99	13.87 ± 3.41	28.17 ± 0.35	4.75 ± 0.72	34.47 ± 0.65
M/CTRL	74.55 ± 2.92	12.64 ± 1.55	16.45 ± 3.22	28.2 ± 0.42.9	4.4 ± 0.99	26.45 ± 2.87
NM/EXP	74.47 ± 1.55	13.95 ± 0.62	0.56 ± 0.41	28.14 ± 0.26	6.24 ± 0.88	31.77 ± 0.33
NM/CTRL	76.05 ± 1.6	11.9 ± 0.88	1.43 ± 0.33	28.11 ± 0.25	6.47 ± 0.7	26.52 ± 1.64

Median ± standard deviation, for age, years of education, years of musical education, MMSE, and GDS. M/EXP, musicians’ improvisation group; M/CTRL, musicians’ imitation group; NM/EXP, non-musicians’ improvisation group; NM/CTRL, non-musicians’ imitation group.

Regarding the socio-demographic information (Table 1), no differences were found between the groups in terms of age *p >* 0.05. Nonetheless, depending on the educational level, there were differences in the Intervention factor *F*(1, 68) = 5.95, *p* = 0.017, $\eta_{p}^{2}$ = 0.084, where the improvisation groups had a higher educational level than the imitation groups. For this reason, educational level was a co-variable in the statistical analyses performed for the memory evaluations of the RCF. As regards musical expertise, there were differences in the Training factor *F*(1, 68) = 61.26, *p* < 0.0001, $\eta_{p}^{2}$ = 0.485, as expected, since we selected musicians and non-musicians for the samples. The average year of musical experience in the musicians’ group was 15.24 ± 2.4 years. Non-musicians had an average musical experience of 0.96 ± 0.3 years.

### Copy of the RCF

**Figure 3:**
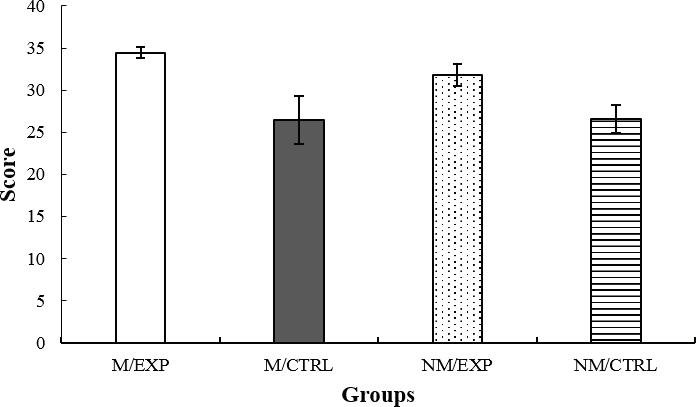
Score for the copy of the RCF. M/EXP: musicians’ improvisation group; M/CTRL: musicians’ imitation group; NM/EXP: non-musicians’ improvisation group; and NM/CTRL: non-musicians’ imitation group.

The acquisition of neutral visual information was evaluated through the copy of the RCF. The results are depicted in Figure 3. The ANCOVA indicated a main effect of the Intervention factor *F*(1, 64) = 9.98, *p* = 0.002, $\eta_{p}^{2}$ = 0.135. The *post hoc* test showed that the improvisation groups had higher copy scores than the imitation groups. Due to this result, copy was implemented as an additional co-variable in the subsequent memory analysis (immediate and deferred).

### Immediate Measures

**Figure 4:**
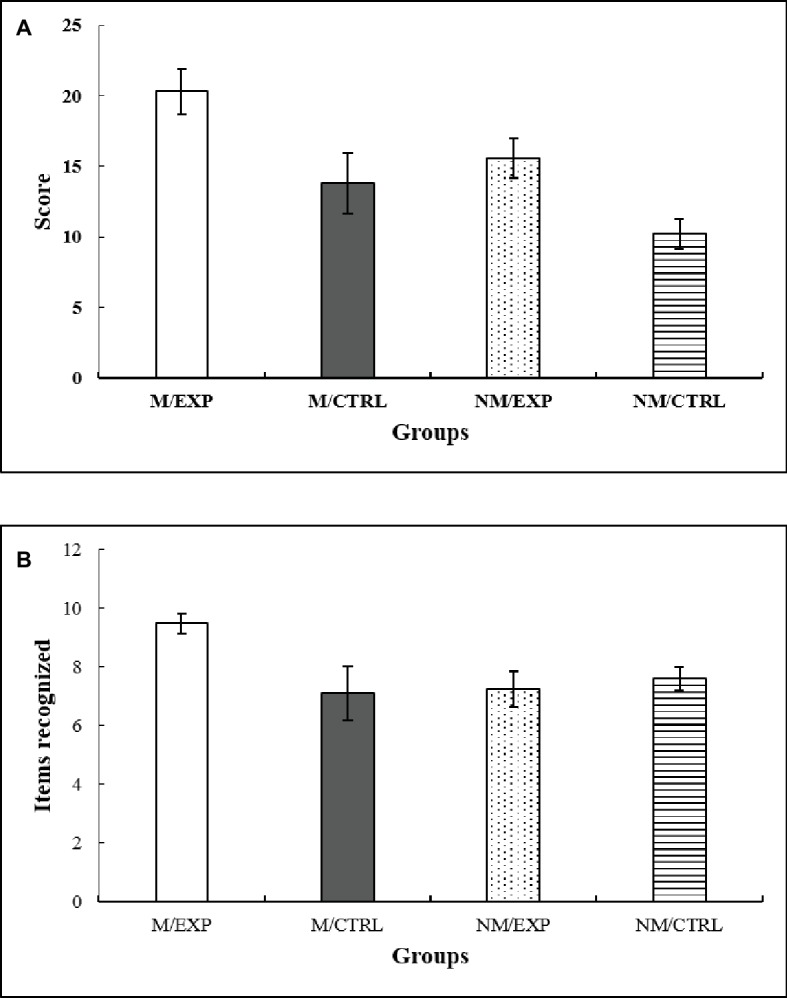
**(A)** Immediate free recall. Score obtained according to the accuracy and location of the figure drawn from memory. **(B)** Immediate recognition. Number of items recognized in a set of 24 items. M/EXP: musicians’ improvisation group; M/CTRL: musicians’ imitation group; NM/EXP: non-musicians’ improvisation group; and NM/CTRL: non-musicians’ imitation group. Vertical lines represent standard errors of the mean.

After being exposed to the different musical interventions, the participants were instructed to draw from memory the RCF that they had seen in the acquisition phase. The ANCOVA yielded a main effect of Training *F*(1, 63) = 8.68, *p* = 0.005, $\eta_{p}^{2}$ = 0.121. The *post hoc* analysis indicated that musicians had a better recall of the RCF than non-musicians (Figure 4A).

Recognition was the second task employed to evaluate memory. The participants watched 24 items, and they had to decide which ones were part of the RCF and which were new. False recognitions were subtracted from the total recognition score. The results are depicted in Figure 4B. The ANCOVA indicated a significant effect of the double interaction Training × Intervention *F*(1, 63) = 4.889, *p* = 0.031, $\eta_{p}^{2}$ = 0.072. The *post hoc* test showed that the musicians’ improvisation group had a better recognition score than non-musicians’ improvisation group. Also, this test indicated that the non-musicians’ imitation group had a better recognition score than the musicians’ imitation group.

### Deferred Measures

**Figure 5:**
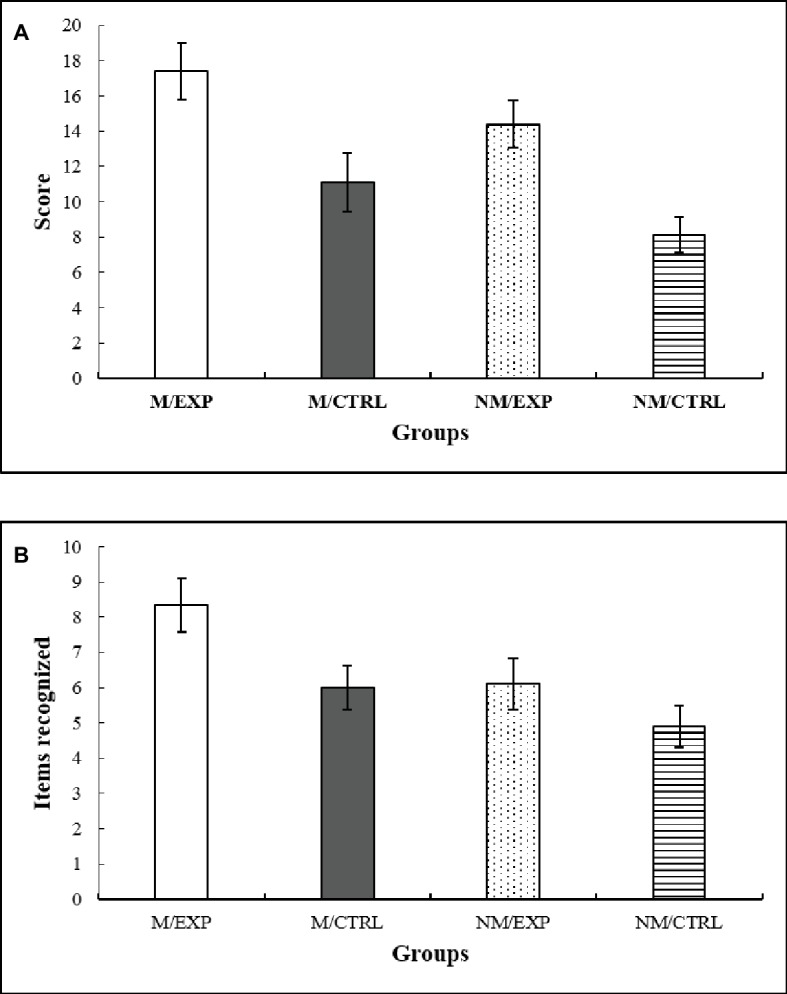
**(A)** Deferred free recall. Score obtained according to the accuracy and location of the figure drawn from memory after 7 days. **(B)** Deferred recognition. Number of components recognized in a set of 24 figures, after 7 days. M/EXP: musicians’ improvisation group; M/CTRL: musicians’ imitation group; NM/EXP: non-musicians’ improvisation group; and NM/CTRL: non-musicians’ imitation group. Vertical lines represent standard errors of the mean.

After 7 days, free recall and recognition were again evaluated (deferred measures). Regarding free recall, the ANCOVA indicated a main effect of Intervention *F*(1, 57) = 8.36, *p* = 0.005, $\eta_{p}^{2}$ = 0.128. The *post hoc* test showed that improvisation groups had a better recall of the RCF than imitation groups (Figure 5A).

After the free recall evaluation, participants watched 24 items, and they had to decide which ones were part of the RCF and which were new. False recognitions were subtracted from the total recognition score (Figure 5B). The ANCOVA yielded a main effect of Training *F*(1, 57) = 4.696, *p* = 0.034, $\eta_{p}^{2}$ = 0.076. The corresponding *post hoc* indicated that the participants with formal musical knowledge had a better recognition score than the non-musicians.

## Study 2

### Materials and Methods

#### Participants

Sixty-three *new* volunteers (76% female) between the ages of 60 and 90 (*M* = 71.94; *SD* = 0.91) participated in this study. Twenty-five were musicians (M) with more than 5 years of musical formal training (schools, institutes, music conservatories). Thirty-eight participants were considered non-musicians (NM). An *a priori* power analysis suggested that *N* = 57 would be adequate to provide 0.60 power (Faul et al., 2007). They were recruited from different senior cultural centers through online announcements. The participant exclusion criteria were the same as those used in Study 1. Each participant signed a written informed consent form and completed a questionnaire where socio-demographic and musical expertise information was requested. The procedure was approved by the University of Buenos Aires Ethics Committee.

#### Measures

##### General Cognitive State Evaluation

This evaluation was conducted in the same way as in Study 1.

##### Emotional Memory Evaluation

The material for the emotional memory task consisted of thirty-six pictures selected from the International Affective Pictures System (IAPS; Lang et al., 1995). Twenty-four pictures were emotionally arousing (12 with a positive valence and 12 with a negative valence) and 12 were non-arousing, neutral images. Following guidelines from previous works (Cahill et al., 2003), we selected the pictures, which covered a wide range of arousal (from 2.95 to 6.36) and valence (from 1.97 to 4.93) in line with the manual by Lang et al. (1995).

#### Instrumental Setting

The setting was the same as the one used in Study 1.

##### Musical Interventions

The musical interventions were the same as the ones used in Study 1.

#### Experimental Design

Because there were two interventions (EXP vs. CTRL) and the participants had different levels of musical expertise (M and NM), a 2(Intervention) × 2(Training) experimental design was run, with four groups with the following number of subjects: (1) M/EXP: musicians’ improvisation group (*n* = 13); (2) M/CTRL: musicians’ imitation group (*n* = 12); (3) NM/EXP: non-musician’ improvisation group (*n* = 18); and (4) NM/CTRL: non-musicians´ imitation group (*n* = 20). Participants were randomly and blindly assigned to the different groups, and they were always tested in groups, with a minimum of four and a maximum of 10 participants in order for the researchers to control the involvement of each participant in the music performance.

##### Procedure

This study was also divided into two sessions with a one-week intersession interval. The first session consisted of four immediately consecutive phases. The first phase was identical to the one used in Study 1.

In the second phase (acquisition phase, about 7 min), the participants watched the 36 selected pictures for 7 s each. The pictures were presented in random order except for the first and last locations in the series, which had to meet the condition of being a neutral picture (Cahill et al., 2003). Simultaneously, the participants were asked to rate on a 0–10 scale “how emotional” or “activating” they felt each image was (from 0 = not arousing at all to 10 = highly arousing). This behavioral task (*Arousal* task) was included in order to (1) ensure that the participants paid attention to each image; (2) validate the selection of IAPS images for this research context, and (3) compare the emotional impact of the images between M-NM groups prior to the musical intervention.

The third phase (intervention) was identical to the one employed in Study 1. Soon afterwards, in the fourth phase (test phase, about 11 min), a two-task test was run. The participants were asked to describe in one word or short phrase as many pictures as they could remember (*Immediate Free Recall* task). Next, they observed the 36 original pictures mixed with 36 new pictures in a random order and they had to mark on a sheet of paper if they had seen the image before or not (*Immediate Recognition* task).

The second session (11 min) was held a week later, when the two-task test was run again (*Deferred Free Recall* task and *Deferred Recognition* task; see Figure 2 for a schematic design of the procedure).

##### Data Analysis

Age, years of formal education, and years of musical education were analyzed independently *via* univariate analysis of variance (ANOVA), where *Intervention* (improvisation vs. imitation) and *Training* (musicians vs. non-musicians) were the between-factors.

Because musicians had more years of education than non-musicians (data shown in Results), arousal, recall, and recognition (immediate and deferred) were independently analyzed *via* repeated measures (RM) ANCOVA with *Intervention* (improvisation vs. reproduction) and *Training* (musicians vs. non-musicians) as the between-factors, *Picture* (neutral, positive, and negative) as the RM, and *Education* as the co-variable.

*Post hoc* least-significant difference (LSD) pairwise comparisons were conducted to analyze significant interactions. The partial Eta square ($\eta_{p}^{2}$) was utilized to estimate effect size. The alpha value was set at 0.05, and the SPSS software package was used to compute descriptive and inferential statistics.

## Results

### Socio-Demographic Characteristics and General Cognitive State

The final sample was composed of 52 participants, because 11 evaluations were discarded due to cognitive impairment or depression; the final number of participants per group was as follows: (1) M/EXP = 12; (2) M/CTRL = 10; (3) NM/EXP = 15; and (4) NM/CTRL = 15. The general cognitive state information (MMSE and GDS) is depicted in Table 2.

**Table 2:** Socio-demographic and general cognitive state data.

Groups	Age	Education	Musical educ.	MMSE	GDS
M/EXP	73 ± 2.32	14.36 ± 0.93	15.36 ± 3.84	28.78 ± 0.43	4.71 ± 0.7
M/CTRL	73 ± 3.28	17.25 ± 2.15	17 ± 6.63	27.67 ± 0.9	4.14 ± 1.06
NM/EXP	71.8 ± 1.72	12.93 ± 1.06	1.07 ± 0.41	28.94 ± 0.25	4.76 ± 0.58
NM/CTRL	71.47 ± 1.59	13.07 ± 0.65	0 ± 0	28.73 ± 0.21	3.47 ± 0.71

Media ± standard deviation, for age, years of education, years of musical education, MMSE, and GDS. M/EXP, musicians’ improvisation group; M/CTRL, musicians’ imitation group; NM/EXP, non-musicians’ improvisation group; and NM/CTRL, non-musicians’ imitation group.

Regarding socio-demographic information (Table 2), there were no differences between groups related to age *p >* 0.05. Nonetheless, there were differences depending on the educational level related to the Training factor *F*(1, 44) = 5.79, *p* = 0.02 $\eta_{p}^{2}$ = 0.116. The musicians had a higher academic level than the non-musicians, and therefore, this variable was considered a co-variable in the statistical analyses that were performed for memory. There were differences in musical level related to the Training factor *F*(1, 45) = 29.53, *p* < 0.0001, $\eta_{p}^{2}$ = 0.39, as expected, since we selected musicians and non-musicians for the samples. The average year of musical experience in the musicians’ group was 16.05 ± 3.43 years. Non-musicians had an average musical experience of 0.53 ± 0.23 years.

### Arousal

**Figure 6:**
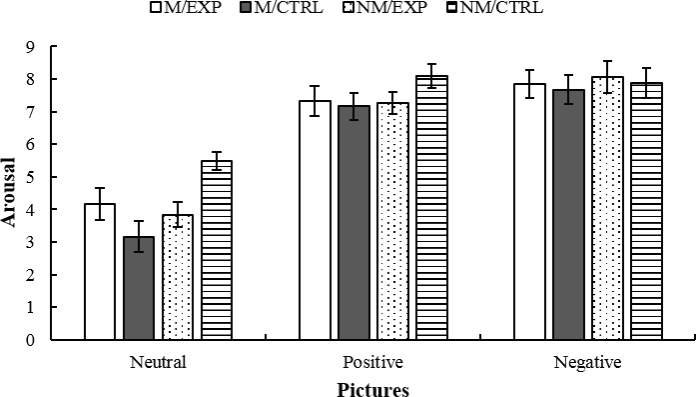
Assessment of neutral, positive, and negative images on a scale from 0 to 10 (0 = non-arousing at all. 10: The highest level of arousal elicited from the participant). M/EXP: musicians’ improvisation group; M/CTRL: musicians’ imitation group; NM/EXP: non-musicians’ improvisation group; and NM/CTRL: non-musicians’ imitation group. Vertical lines represent standard errors of the mean.

Arousal was the first dependent variable analyzed. Participants watched neutral, positive, and negative images, and simultaneously rated, from 0 to 10, how arousing the pictures were for them. The emotional pictures were rated as more activating than the neutral ones, and the rating of neutral images was affected by Training and Intervention (Figure 6). These impressions were corroborated by the statistical analysis, since the ANCOVA yielded a main effect of Picture *F*(2, 86) = 12.817, *p* < 0.0001, $\eta_{p}^{2}$ = 0.230, while the corresponding *post hoc* indicated that the emotional images were considered more activating than the neutral ones. Besides, the effect of the Picture × Intervention interaction was significant *F*(1, 43) = 5.23, *p* = 0.027, $\eta_{p}^{2}$ = 0.108, and the triple interaction Picture × Intervention × Training was also significant *F*(2, 86) = 4.27, *p* = 0.017, $\eta_{p}^{2}$ = 0.09. The analysis of the triple interaction indicated that the M/EXP group rated the neutral images as more activating than did the M/CTRL group, while the opposite pattern was observed in non-musicians since the NM/CTRL group rated the neutral images as more activating than did the NM/EXP group. In addition, the NM/CTRL group rated the neutral images as more activating than did the M/CTRL group.

### Immediate Measures

**Figure 7:**
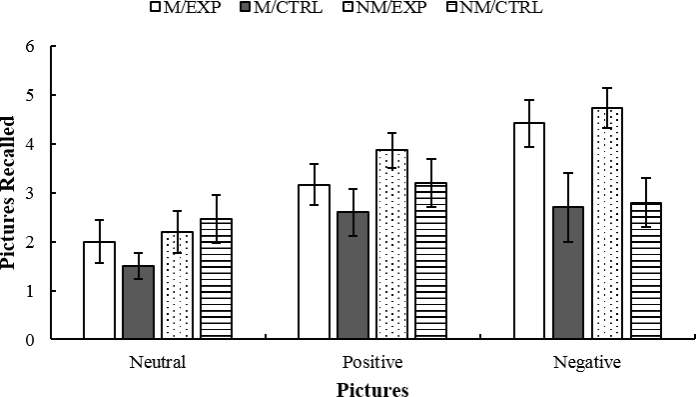
Immediate free recall. Mean number of neutral, positive, and negative pictures that groups could remember after the treatment. M/EXP: musicians’ improvisation group; M/CTRL: musicians’ imitation group; NM/EXP: non-musicians’ improvisation group; and NM/CTRL: non-musicians’ imitation group. Vertical lines represent standard errors of the mean.

After participants were exposed to the intervention (imitation or improvisation), they were asked to recall as many pictures as they could. The ANCOVA indicated a significant effect of Intervention *F*(1, 43) = 6.93, *p* = 0.012, $\eta_{p}^{2}$ = 0.139, where the *post hoc* showed that the improvisation group remembered more images than the imitation group. Also, the double interaction Picture × Intervention achieved significance *F*(2, 86) = 5.22, *p* = 0.007, $\eta_{p}^{2}$ = 0.108. The *post hoc* indicated that the improvisation group remembered more negative images than the imitation group. The results are depicted in Figure 7.

After the free recall, the participants observed the 36 original pictures randomly intermixed with 36 new ones. They had to discriminate the new images from the old ones. The ANCOVA indicated no significant differences in Training, Picture, or Intervention, or any of their interactions, *p* > 0.05 (data not shown).

### Deferred Measures

**Figure 8:**
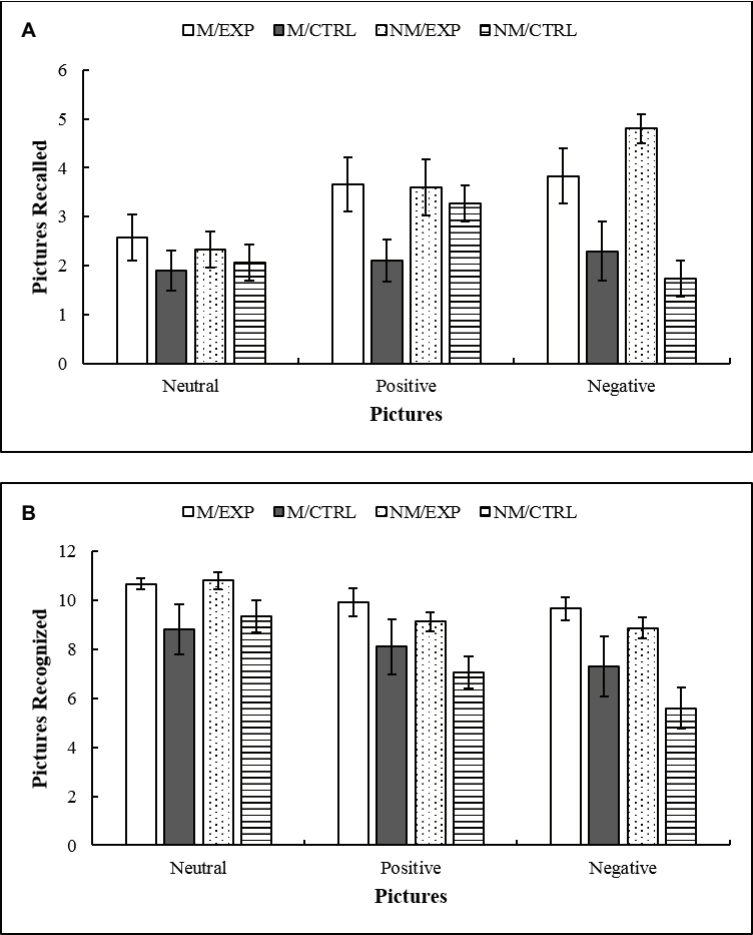
**(A)** Deferred free recall. Mean number of neutral, positive, and negative pictures that groups could remember after a week, between treatment and testing. **(B)** Deferred recognition. Number of neutral, positive, and negative pictures that participants could recognize as previously seen from a pool of 72 images. M/EXP: musicians’ improvisation group; M/CTRL: musicians’ imitation group; NM/EXP: non-musicians’ improvisation group; and NM/CTRL: non-musicians’ imitation group. Vertical lines represent standard errors of the mean.

The test of free recall and recognition tasks was repeated a week later. Figure 8A illustrates the results of the free recall task. The ANCOVA indicated a main effect of Intervention *F*(1, 43) = 18.27, *p* < 0.0001, $\eta_{p}^{2}$ = 0.29, the *post hoc* showed that the improvisation groups remembered more images than the imitation groups. The double interaction Picture × Intervention also achieved significance *F*(2, 86) = 5.59, *p* < 0.005, $\eta_{p}^{2}$ = 0.115, and the corresponding *post hoc* indicated that for positive and negative images the improvisation groups remembered more images than the imitation groups.

To evaluate recognition, the 36 target pictures were mixed with 36 new pictures and participants had to indicate whether the images were new or old (Figure 8B). False recognitions were subtracted from the total recognition score (from each of the pictures). The ANCOVA showed a significant main effect of Intervention *F*(1, 43) = 9.76, *p* = 0.003, $\eta_{p}^{2}$ = 0.185, where the improvisation groups recognized more images than the imitation groups. In addition, there was a main effect of Picture *F*(2, 86) = 3.17, *p* = 0.047, $\eta_{p}^{2}$ = 0.069, and the *post hoc* indicated that the neutral pictures had a better recognition score than the positive and negative ones and also that the positive images were better recognized than the negative ones. Finally, the double interaction Picture × Intervention achieved significance *F*(2, 86) = 3.29, *p* = 0.042, $\eta_{p}^{2}$ = 0.071. This interaction indicated that in the three types of images, the improvisation group had a better recognition score than the imitation groups.

## Discussion

The goal of this work was to evaluate if a musical intervention could improve neutral or emotional memory in older adults with or without formal musical knowledge. Our control group was not a passive one; instead, it participated in a group musical activity, allowing us to detect specific parameters in each type of intervention that could explain the possible benefits of improvisation. The main results indicated that musical improvisation enhanced memory especially when the information to be consolidated was emotional, indicating that the intervention is more linked to the emotional content than to the neutral one. In addition, musicians performed better than non-musicians. In the following paragraphs, each of the findings is explained in detail.

In both studies, the improvisation groups had a better mnemonic performance than the imitation groups. Nonetheless, this effect was higher in Study 2, where memory with emotional content was evaluated. The improvisation groups performed better at their immediate and deferred free recall and also at their deferred recognition than the imitation groups. Furthermore, in the immediate free recall, the negative images were better remembered; in the deferred free recall, both positive and negative images; and in the deferred recognition, the three types of images were better recognized. In other words, over time, the information had a better consolidation and the participants remembered or recognized more information. By contrast, in the complex figure, better performance was achieved in the improvisation condition only for the deferred free recall. These results would indicate that there was an interaction between musical improvisation and visual memory, and the greatest effect was found for the emotion-laden information.

A possible explanation for these findings is that during the experience of musical improvisation a melody and a rhythm are spontaneously created, integrating the emotional with the different cognitive levels (Bruscia, 1998, 1999). In this musical technique, all the body is used to express intentions, emotions, and memories. For this reason, musical improvisation is defined as a special self-expression technique (Gilboa et al., 2006; Punkanen, 2011; Godman, 2012; McPherson et al., 2014). Besides, it has been shown that sound is a potent elicitor of emotions and that musical experiences activate specific pathways in several brain areas associated with emotional content, such as the cingulate and insular cortices, hypothalamus, hippocampus, amygdala, and prefrontal cortex (Boso et al., 2006; Koelsch, 2012, 2014). A study conducted by Koelsch et al. (2018) demonstrated that the auditory cortex, activated during the musical perception, hosts regions that are influential within networks underlying the affective processing of auditory information. The emotional state induced by the musical improvisation may have enhanced the emotions produced by the affective pictures, thus strengthening the memory process. Some studies indicate that music, because of the emotionality state that it generates (Koelsch, 2012), will work as an enhancer of visual elements loaded with emotion (Logeswaran and Bhattacharya, 2009; Kamiyama et al., 2013), causing a synergy between both emotional states. In the first study, this synergic effect between the emotion aroused by the improvisation and the emotion aroused by the task was not observed, probably because the stimuli lacked emotional content.

Musical improvisation, as opposed to imitation where a pattern is replicated, is characterized by the presence of creative elements. This characteristic would indicate that it is not the music itself that modulates memory, since in the imitation condition, participants also perceive and produce musical components but rather the creation of a novel musical product in groups. In future studies, a creative non-musical group could be added to address this topic. Besides, spontaneous improvisation, as opposed to the performance of learned sequences (as in the imitation), is characterized by an extensive deactivation of the medial dorsolateral prefrontal cortex and lateral orbital regions with a focal activation of the medial prefrontal cortex (Limb and Braun, 2008). In addition, there is a relation between musical improvisation and autobiographic memories, since independently of the level of complexity used in the improvisation, the prefrontal and medial temporal cortices are activated, and these areas are involved in memory (Limb and Braun, 2008).

Imitation could interfere with memory. When there are restrictions, especially attentional ones where the participant is asked to replicate, repeat a pattern, adjust to it in intensity, and synchronize, this intervention could diminish cognitive resources and lead to mnemonic deterioration (Miendlarzewska et al., 2013). This is relevant since most musical activities designed for older adults are repetitive (the typical case is the choir, where the participant has to memorize his or her part, pay attention to the tuning, rhythm, etc.). Even though these activities reinforce musical contents *per se*, they are less efficient when the goal is to improve cognitive skills such as memory.

In the first study, an effect of musicianship was found, which is in line with previous studies about the effect of musical training on visual memory (Hanna-Pladdy and MacKay, 2011). Musicians outperformed non-musicians in immediate free recall and recognition and in deferred recognition. A plausible explanation for the better performance of musicians is that there are structural and functional brain differences between musicians and non-musicians (Zatorre, 1998; Gaser and Schlaug, 2003; Lotze et al., 2003; Bermúdez and Zatorre, 2005; Zatorre et al., 2007; Justel and Diaz Abrahan, 2012; Barrett et al., 2013; Strait and Kraus, 2014; Schlaug, 2015; Herrero and Carriedo, 2018; Li et al., 2018). Becoming a skilled musician requires extensive training, and the type of learning involved entails the development of several abilities (e.g., perception, cognitive control, memory, motor skills, among others). The abilities developed by musicians induce connections and interactions between several brain areas. The brain structural differences between musicians and non-musicians were found to involve the enlargement or thickening of numerous areas in people with musical training. Some of these differences were associated with the anteromedial portion of Heschl’s gyrus, the corpus callosum, the *planum temporale*, and with changes in gray matter that implied a greater plasticity (Luders et al., 2004; Bermúdez et al., 2009; Anaya et al., 2016).

At the same time, the structural differences are accompanied by functional and behavioral divergences in several domains (Herrero and Carriedo, 2018). Depending on the extent of the effect of musical training, the near transfer label is used when the cognitive functions affected by training are those related closely with music, such as the recognition of melodic contour or intervallic sequences (Fujioka et al., 2004). While musical training could transfer cognitive advantages that go beyond musical areas, if the functional change is observed in non-musical skills such as language (Schlaug et al., 2005), mathematical reasoning (Vaughn, 2000), or attentional functions (Wang et al., 2015), the process is named far transfer. In the present work, we contribute evidence to the far-transfer literature, since the benefits for musicians were observed in a cognitive skill not strictly related to musical training.

The fact that we found no differences in terms of musical training in the second study could be associated with non-musicians benefiting from the information with emotional content, and accordingly the greater effect was observed in the intervention factor (improvisation vs. imitation). Because, in the first study, the emotional components were not present, the prevailing factor was musicianship (training). Therefore, the emotionality effect associated with the intervention (improvisation) could have shadowed the training factor effects in the second study.

Nonetheless, it is not necessary to be a professional musician and have lifetime experience in music to benefit from musical training. Some studies indicated that only 1 week of stimulation in musical perception and production resulted in functional changes in the participants (Bangert and Altenmüller, 2003). Besides, it has been demonstrated that older adults who began their musical training in old age had benefits in several cognitive domains (Bugos et al., 2007). Thus, focal musical interventions (such as the one proposed in the present work) as well as short- and long-term interventions induced a benefit in the cognitive functions of older age participants.

Studies about the effect of music in visual memory are scarce. As far as we know, no research has so far focused on memory with emotional content, and it is in this topic that the novelty of our study lies. Besides, the relation between musical experience and neutral visual memory has been the topic of few studies, with conflicting results. Fauvel et al. (2014) found no enhancement of neutral memory in older adults. However, in agreement with our results, Hanna-Pladdy and Mackay (2011) found an improvement in the visual memory of musicians compared to non-musicians. Notably, as far as learning and evaluation of memory are concerned, there are different tests to evaluate this cognitive function, and it is precisely this issue that differentiates the mentioned studies. The methodologies used for measuring memory could have resulted in the divergences found in the studies.

The limitations of our study involve the inclusion criterion to be considered a musician. The criterion was to have more than 5 years of musical training, and although the participants were asked what musical instrument they played, they were not asked whether they were currently active, how many hours a week they devoted to musical training, or how old they were when they started learning music. These questions will be included in future research. In addition, although we found differences regarding the educational level, this variable was used as a co-variable in the statistical analyses so as not to bias the results. Another limitation of our studies is the sampling. In both studies, more than half of the samples were women, and it is possible that the effects might vary across genders, given that some studies show female participants to be more receptive to emotional cues (Andreano et al., 2008; Nielsen et al., 2011, 2013; Felmingham et al., 2012). We intend to improve this point in future research.

A key challenge for successful aging is to discover cognitive treatments or interventions that have the ability to integrate multiple neural systems that alleviate or prevent age-related cognitive decline (Bugos et al., 2007). Making music is the optimal cognitive intervention that includes multimodal sensorimotor integration, creation of novel elements, motivation, and difficulty. It is relevant to highlight the difference between improvisation and imitation, since the standard musical activities for older adults involve repetitive tasks with no novelty component. By endorsing the advantages of improvisation, group activities could be designed for the purpose of creating something musically novel in a context of social interaction. In addition, improvisation being a social practice, it increases the adherence to the treatment diminishing the dropout rates. Besides, this musical intervention had the benefit that it was pleasant, a motivational factor for the participant to perform this kind of activity, as opposed to other types of training. As a result, despite being a focal intervention, it could be presented in a regular schedule, since the core component is the creation of something musical and always novel. As music improvisation modulates memory, music treatment may provide a simple, safe, and effective method of preventing the potentially harmful physiological concomitants of memory impairment, with great potential for clinical application.

## Ethics Statement

The participants of the studies gave voluntary written consent to take part in the studies without obtaining any type of remuneration and according to the requirements of The Declaration of Helsinki.

## Author Contributions

VDA and NJ contributed to the conception and design of the studies. VDA conducted the studies. VDA and NJ contributed to data analysis. VDA, FS, and NJ participated in the writing of the paper and interpretation of the data. FS and NJ supervised and integrated the information.

### Conflict of Interest Statement

The authors declare that the research was conducted in the absence of any commercial or financial relationships that could be construed as a potential conflict of interest.

The handling editor declared a shared affiliation, though no other collaboration, with several of the authors VDA and NJ at time of review.

## References

[ref1] Abutalebi J. Guidi G. Borsa V. Canini M. Della Rosa P. Parris B.. (2015). Bilingualism provides a neural reserve for aging populations. Neuropsychologia 69, 201–210. 10.1016/j.neuropsychologia.2015.01.040, PMID: 25637228

[ref2] Amer T. Kalender B. Hasher L. Trehub S. E. Wong Y. (2013). Do older professional musicians have cognitive advantages? PLoS One 8:e71630. 10.1371/journal.pone.0071630, PMID: 23940774 PMC3737101

[ref3] Anaya E. Pisoni D. Kronenberger W. (2016). Long-term musical experience and auditory and visual perceptual abilities under adverse conditions. J. Acoust. Soc. Am. 140, 2074–2081. 10.1121/1.4962628, PMID: 27914434 PMC5734909

[ref4] Andreano J. Arjomandi H. Cahill L. (2008). Menstrual cycle modulation of the relationship between cortisol and long-term memory. Psychoneuroendocrinology 33, 874–882. 10.1016/j.psyneuen.2008.03.009 18468808

[ref5] Bangert M. Altenmüller E. O. (2003). Mapping perception to action in piano practice: a longitudinal DC-EEG study. Neuroscience 4:26. 10.1186/1471-2202-4-26, PMID: 14575529 PMC270043

[ref6] Barrett K. Ashley R. Strait D. Kraus N. (2013). Art and science: how musical training shapes the brain. Front. Psychol. 16:713. 10.3389/fpsyg.2013.00713 PMC3797461 24137142

[ref7] Bengtsson S. Csikszenymihalyi M. Ullén F. (2007). Cortical regions involved in the generation of musical structures during improvisation in pianists. J. Cogn. Neurosci. 19, 830–842. 10.1162/jocn.2007.19.5.830, PMID: 17488207

[ref8] Berkowitz A. Ansari D. (2008). Generation of novel motor sequences: the neural correlates of musical improvisation. NeuroImage 41, 535–543. 10.1016/j.neuroimage.2008.02.028, PMID: 18420426

[ref9] Berkowitz A. Ansari D. (2010). Expertise-related deactivation of the right temporoparietal junction during musical improvisation. NeuroImage 49, 712–719. 10.1016/j.neuroimage.2009.08.042, PMID: 19715764

[ref10] Bermúdez P. Lerch J. Evans A. Zatorre R. (2009). Neuroanatomical correlates of musicianship as revealed by cortical thickness and voxel-based morphometry. Cereb. Cortex 19, 1583–1596. 10.1093/cercor/bhn196, PMID: 19073623

[ref11] Bermúdez P. Zatorre R. (2005). Differences in gray matter between musicians and nonmusicians. Ann. N. Y. Acad. Sci. 1060, 395–399. 10.1196/annals.1360.057, PMID: 16597791

[ref12] Bermúdez-Rattoni F. Prado-Alcalá R. (2001). Memoria. ¿En dónde está y cómo se forma? (México: Editorial Trillas).

[ref13] Boso M. Politi P. Barale F. Emanuele E. (2006). Neurophysiology and neurobiology of the musical experience. Funct. Neurol. 21, 187–191., PMID: 17367577

[ref14] Bottiroli S. Rosi A. Russo R. Vecchi T. Cavallini E. (2014). The cognitive effects of listening to background music on older adults: processing speed improves with upbeat music, while memory seems to benefit from both upbeat and downbeat music. Front. Aging Neurosci. 6:284. 10.3389/fnagi.2014.00284, PMID: 25360112 PMC4197792

[ref15] Bruscia K. (1998). Musicoterapia. Métodos y prácticas. (México: Editorial Pax México).

[ref16] Bruscia K. (1999). Modelos de improvisación en musicoterapia. (España: Agruparte Victoria –Gasteiz).

[ref17] Bugos J. A. Perlstein W. M. McCrae C. S. Brophy T. S. Bedenbaugh P. H. (2007). Individualized piano instruction enhances executive functioning and working memory in older adults. Aging Ment. Health 11, 464–471. 10.1080/13607860601086504, PMID: 17612811

[ref18] Butman J. Arizaga R. L. Harris P. Dranke M. Baumann D. de Pascale A. (2001). El “mini - mental state examination” en español. Normas para Buenos Aires. Rev. Neurol. Arg. 26, 11–15.

[ref19] Cahill L. Gorski L. Le K. (2003). Enhanced human memory consolidation with post-learning stress: interaction with the degree of arousal at encoding. Learn. Mem. 10, 270–274. 10.1101/lm.62403, PMID: 12888545 PMC202317

[ref20] Cahill L. McGaugh J. L. (1995). A novel demonstration of enhanced memory associated with emotional arousal. Conscious. Cogn. 4, 410–421. 10.1006/ccog.1995.1048, PMID: 8750416

[ref21] Christie G. J. Hamilton T. Manor B. D. Farb N. A. S. Farzan F. Sixsmith A. (2017). Do lifestyle activities protect against cognitive decline in aging? A Review. Front. Aging Neurosci. 9:381. 10.3389/fnagi.2017.00381 PMC5701915 29209201

[ref22] Diaz Abrahan V. Justel N. (2015). La improvisación musical. Una mirada compartida entre la musicoterapia y las neurociencias. Psicogente 18, 372–384. 10.17081/psico.18.34.512

[ref23] Fancourt D. Ockelford A. Belai A. (2014). The psychoneuroimmunological effects of music: a systematic review and a new model. Brain Behav. Immun. 36, 15–26. 10.1016/j.bbi.2013.10.014, PMID: 24157429

[ref24] Faul F. Erdfelder E. Lang A. Buchner A. (2007). G* Power 3: a flexible statistical power analysis program for the social, behavioral, and biomedical sciences. Behav. Res. Methods 39, 175–191. 10.3758/BF03193146, PMID: 17695343

[ref25] Fauvel B. Groussard M. Mutlu J. Arenaza-Urquijo E. M. Eustache F. Desgranges B.. (2014). Musical practice and cognitive aging: two cross-sectional studies point to phonemic fluency as a potential candidate for a use-dependent adaptation. Front. Aging Neurosci. 6:227. 10.3389/fnagi.2014.00227, PMID: 25346684 PMC4191346

[ref26] Felmingham K. Tran T. Fong W. Bryant R. (2012). Sex differences in emotional memory consolidation: the effect of stress-induced salivary alpha-amylase and cortisol. Biol. Psychol. 89, 539–544. 10.1016/j.biopsycho.2011.12.006 22248928

[ref27] Ferreri L. Aucouturier J.-J. Muthalib M. Bigand E. Bugaiska A. (2013). Music improves verbal memory encoding while decreasing prefrontal cortex activity: an fNIRS study. Front. Hum. Neurosci. 7:779. 10.3389/fnhum.2013.00779, PMID: 24339807 PMC3857524

[ref28] Folstein M. F. Folstein S. E. McHugh P. R. (1975). “Mini-mental state”: a practical method for grading the cognitive state of patients for the clinician. J. Psychiatr. Res. 19, 189–198. 10.1016/0022-3956(75)90026-6 1202204

[ref29] Friedman D. (2013). The cognitive aging of episodic memory: a view based on the event-related brain potential. Front. Behav. Neurosci. 26:111. 10.3389/fnbeh.2013.00111 PMC3752587 23986668

[ref30] Fujioka T. Trainor L. Ross B. Kakigi R. Pantev C. (2004). Musical training enhances automatic encoding of melodic contour and interval structure. J. Cogn. Neurosci. 16, 1010–1021. 10.1162/0898929041502706, PMID: 15298788

[ref31] Gaser C. Schlaug G. (2003). Brain structures differ between musicians and non-musicians. J. Neurosci. 23, 9240–9245. 10.1523/JNEUROSCI.23-27-09240.2003, PMID: 14534258 PMC6740845

[ref32] Gilbertson S. (2013). Improvisation and meaning. Int. J. Qual. Stud. Health Well Being 7:20604. 10.3402/qhw.v8i0.20604 PMC3740602 23930989

[ref33] Gilboa A. Bodner E. Amir D. (2006). Emotional communicability in improvised music: the case of music therapists. J. Music. Ther. 43, 198–225. 10.1093/jmt/43.3.198, PMID: 17037951

[ref34] Godman A. (2012). “What does one know when one knows how to improvise?” in Proceedings of the 12th international conference on music perception and cognition and the 8th triennial conference of the European society for the cognitive sciences. eds. Cambouropoulos E. Tsougras C. Mavromatis P. Pastiadis K.

[ref35] Hanna-Pladdy B. Gajewski B. (2012). Recent and past musical activity predicts cognitive aging variability: direct comparison with general lifestyle activities. Front. Hum. Neurosci. 6:198. 10.3389/fnhum.2012.00198, PMID: 22833722 PMC3400047

[ref36] Hanna-Pladdy B. MacKay A. (2011). The relation between instrumental musical activity and cognitive aging. Neuropsychology 25, 378–386. 10.1037/a0021895, PMID: 21463047 PMC4354683

[ref37] Herrero L. Carriedo N. (2018). Differences in updating processes between musicians and non-musicians from late childhood to adolescence. Learn. Individ. Differ. 61, 188–195. 10.1016/j.lindif.2017.12.006

[ref38] Iuliano E. di Cagno A. Aquino G. Fiorilli G. Mignogna P. Calcagno G.. (2015). Effects of different types of physical activity on the cognitive functions and attention in older people: a randomized controlled study. Exp. Gerontol. 70, 105–110. 10.1016/j.exger.2015.07.008, PMID: 26183691

[ref39] Judde S. Rickard N. (2010). The effect of post-learning presentation of music on long term word list retention. Neurobiol. Learn. Mem. 94, 13–20. 10.1016/j.nlm.2010.03.002, PMID: 20307678

[ref40] Justel N. Diaz Abrahan V. (2012). Plasticidad cerebral: participación del entrenamiento musical. Suma Psicol. 19, 97–108. 10.14349/sumapsi2012.1234

[ref42] Kamiyama K. Abla D. Iwanaga K. Okanoya K. (2013). Interaction between musical emotion and facial expression as measured by event-related potentials. Neuropsychologia 51, 500–505. 10.1016/j.neuropsychologia.2012.11.031, PMID: 23220447

[ref43] Kämpfe J. Sedlmeier P. Renkewitz F. (2010). The impact of background music on adult listeners: a meta-analysis. Psychol. Music 39, 424–448. 10.1177/0305735610376261, PMID: 21119751

[ref44] Koelsch S. (2012). Brain and Music. (West Sussex: Wiley-Blackwell).

[ref45] Koelsch S. (2014). Brain correlates of music-evoked emotions. Nat. Rev. Neurosci. 15, 170–180. 10.1038/nrn3666, PMID: 24552785

[ref46] Koelsch S. Skouras S. Lohmann G. (2018). The auditory cortex hosts network nodes influential for emotion processing: an fMRI study on music-evoked fear and joy. PLoS One 13:22. 10.1371/journal.pone.0190057 PMC5791961 29385142

[ref47] Kramer A. F. Bherer L. Colcombe S. J. Dong W. Greenough W. T. (2004). Environmental influences on cognitive and brain plasticity during aging. J. Gerontol. 59A, 940–957. 10.1093/gerona/59.9.M940 15472160

[ref49] Lang P. J. Bradley M. M. Cuthbert B. N. (1995). “International affective picture system (IAPS): affective ratings of pictures and instruction manual” in Technical report A-6 (Gainesville, FL: NIMH, Center for the Study of Emotion and Attention).

[ref50] Lappe C. Herholz C. Trainor L. Pantev C. (2008). Cortical plasticity induced by short-term unimodal and multimodal musical training. J. Neurosci. 28, 9632–9639. 10.1523/JNEUROSCI.2254-08.2008 18815249 PMC6671216

[ref51] Li Q. Wang X. Wang Yongqi X. Xie Y. Li X.. (2018). Musical training induces functional and structural auditory-motor network plasticity in young adults. Hum. Brain Mapp. 39, 2098–2110. 10.1002/hbm.23989, PMID: 29400420 PMC6866316

[ref52] Limb C. Braun A. (2008). Neural substrates of spontaneous musical performance: An fMRI study of jazz improvisation. PLoS One 3:e1679. 10.1371/journal.pone.0001679, PMID: 18301756 PMC2244806

[ref53] Logeswaran N. Bhattacharya J. (2009). Crossmodal transfer of emotion by music. Neurosci. Lett. 455, 129–133. 10.1016/j.neulet.2009.03.044, PMID: 19368861

[ref54] Loprinzi P. D. Edwards M. K. Crush E. Ikuta T. Del Arco A. (2018). Dose-response association between physical activity and cognitive function in a national sample of older adults. Am. J. Health Promot. 32, 554–560. 10.1177/0890117116689732 29214828

[ref55] Lotze M. Scheler G. Tan H. R. Braun C. Birbaumer N. (2003). The musician’s brain: functional imaging of amateurs and professionals during performance and imagery. NeuroImage 20, 1817–1829. 10.1016/j.neuroimage.2003.07.018, PMID: 14642491

[ref56] Luders E. Gaser C. Jancke L. Schlaug G. (2004). A voxel-based approach to gray matter asymmetries. NeuroImage 22, 656–664. 10.1016/j.neuroimage.2004.01.032, PMID: 15193594

[ref57] Mammarella N. Fairfield B. Cornoldi C. (2007). Does music enhance cognitive performance in healthy older adults? The Vivaldi effect. Aging Clin. Exp. Res. 19, 394–399. 10.1007/bf03324720, PMID: 18007118

[ref58] Manzano O. Ullén F. (2012). Goal-independent mechanisms for free response generation: creative and pseudo-random performance share neural substrates. NeuroImage 59, 772–780. 10.1016/j.neuroimage.2011.07.016, PMID: 21782960

[ref59] Martinez de la Iglesia J. Onis Vilches C. Dueñas Herrero R. Colomer C. Aguado-Tabernè C. Luque-Luque R. (2002). Versión española del cuestionario de Yesavage abreviado (GDS) para el despistaje de depresión en mayores de 65 años: adaptación y validación. Medifam 12, 620–630. 10.4321/S1131-57682002001000003

[ref60] McPherson M. Lopez-Limb M. Rankin S. Limb C. (2014). The role of emotion in musical improvisation: an analysis of structural features. PLoS One 21:e105144. 10.1371/journal.pone.0105144, PMID: 25144200 PMC4140734

[ref61] Meyers J. E. Meyers K. R. (1995). Rey complex figure test and recognition trial: Psychological Assessment Resources, Inc.

[ref62] Miendlarzewska E. Elswijk G. Cannistraci C. van Ee R. (2013). Working memory load attenuates emotional enhancement in recognition memory. Front. Psychol. 4:112. 10.3389/fpsyg.2013.00112 PMC3600573 23515565

[ref63] Moayeri S. Cahill L. Jin I. Potkin S. (2010). Relative sparing of emotionally influenced memory in Alzheimer's disease. Neuroreport 11, 653–655. 10.1097/00001756-200003200-00001 10757495

[ref64] Nielsen S. Ertman N. Lakhani Y. Cahill L. (2011). Hormonal contraception usage is associated with altered memory or an emotional story. Neurobiol. Learn. Mem. 96, 378–384. 10.1016/j.nlm.2011.06.013 21740976 PMC3148336

[ref65] Nielsen S. Segal S. Worden I. Yim I. Cahill L. (2013). Hormonal contraception use alters stress responses and emotional memory. Biol. Psychol. 92, 257–266. 10.1016/j.biopsycho.2012.10.007, PMID: 23131613 PMC3558603

[ref66] Nyberg L. Sandblom J. Jones S. Neely A. S. Petersson K. M. Ingvar M.. (2003). Neural correlates of training related memory improvement in adulthood and aging. Proc. Natl. Acad. Sci. 100, 13728–13733. 10.1073/pnas.1735487100, PMID: 14597711 PMC263881

[ref67] Pantev C. Herholz S. (2011). Plasticity of the human auditory cortex related to musical training. Neurosci. Biobehav. Rev. 35, 2140–2154. 10.1016/j.neubiorev.2011.06.010, PMID: 21763342

[ref68] Park D. C. Festini S. B. (2017). Theories of memory and aging: a look at the past and a glimpse of the future. J. Gerontol. Ser. B Psychol. Sci. Soc. Sci. 72, 82–90. 10.1093/geronb/gbw066 27257229 PMC5156492

[ref70] Pinho A. Ullén F. Castelo-Branco M. Fransson P. de Manzano O. (2016). Addressing a paradox: dual strategies for creative performance in introspective and extrospective networks. Cereb. Cortex 26, 3052–3063. 10.1093/cercor/bhv130, PMID: 26088973

[ref71] Punkanen M. (2011). “Improvisational music therapy and perception of emotions in music by people with depression” in Jyväskylä studies in humanities 153 (Jyväskylä: University of Jyväskylä), 60 p. (94 p.).

[ref72] Rickard N. Wing Wong W. Velik L. (2012). Relaxing music counters heightened consolidation of emotional memory. Neurobiol. Learn. Mem. 97, 220–228. 10.1016/j.nlm.2011.12.005, PMID: 22207009

[ref73] Schlaug G. (2015). Musicians and music making as a model for the study of brain plasticity. Prog. Brain Res. 217, 37–55. 10.1016/bs.pbr.2014.11.020 25725909 PMC4430083

[ref74] Schlaug G. Norton A. Overy K. Winner E. (2005). Effects of music training on the child’s brain and cognitive development. Ann. N. Y. Acad. Sci. 1060, 219–230. 10.1196/annals.1360.015, PMID: 16597769

[ref75] Schneider C. E. Hunter E. G. Bardach S. H. (2018). Potential cognitive benefits from playing music among cognitively intact older adults: a scoping review. J. Appl. Gerontol. 1:733464817751198. 10.1177/0733464817751198 29361873

[ref76] Sheikh J. I. Yesavage J. A. (1986). Geriatric depression scale (GDS): recent evidence and development of a shorter version. Clin. Gerontol. 5, 165–173. 10.1300/J018v05n01_09

[ref77] Squire L. R. Wixted J. T. (2011). The cognitive neuroscience of human memory since H.M. Annu. Rev. Neurosci. 34, 259–288. 10.1146/annurev-neuro-061010-113720 21456960 PMC3192650

[ref78] Strait D. L. Kraus N. (2014). Biological impact of auditory expertise across the life span: musicians as a model of auditory learning. Hear. Res. 308, 109–121. 10.1016/j.heares.2013.08.004, PMID: 23988583 PMC3947192

[ref79] Talamini F. Altoè G. Carretti B. Grassi M. (2018). Correction: musicians have better memory than nonmusicians: a meta-analysis. PLoS One 13:e0191776. 10.1371/journal.pone.0191776, PMID: 29352315 PMC5774829

[ref80] Thaut M. Gardiner J. Holmberg D. Horwitz J. Kent L. Andrews G. (2009). Neurologic music therapy improves executive function and emotional adjustment in traumatic brain injury rehabilitation. Ann. N. Y. Acad. Sci. 1169, 406–416. 10.1111/j.1749-6632.2009.04585.x 19673815

[ref81] Thaut M. Hoemberg V. (2014). Handbook of neurologic music therapy. (United Kingdom: Oxford University Press).

[ref82] Thompson R. G. Moulin C. Hayre S. Jones R. W. (2006). Music enhances category fluency in healthy older adults and Alzheimer's disease patients. Exp. Aging Res. 31, 91–99. 10.1080/03610730590882819 15842075

[ref83] Tulving E. (2002). Episodic memory: from mind to brain. Annu. Rev. Psychol. 53, 1–25. 10.1146/annurev.psych.53.100901.135114, PMID: 11752477

[ref84] Vaughn K. (2000). Music and mathematics: modest support for the oft-claimed relationship. J. Aesthet. Edu. 34, 149–166. 10.2307/3333641

[ref85] Wang X. Ossher L. Reuter-Lorenz P. A. (2015). Examining the relationship between skilled music training and attention. Conscious. Cogn. 36, 169–179. 10.1016/j.concog.2015.06.014, PMID: 26160137

[ref86] Wigram T. (2004). Improvisation: Methods and techniques for music therapy clinicians, educators, and students. (England: Jessica Kingsley Publishers).

[ref87] World Health Organization (2012). World Health Organization dementia: A public health priority. Available from: https://www.who.int/mental_health/publications/dementia_report_2012/en/

[ref88] Zatorre R. (1998). Functional specialization of human auditory cortex for musical processing. Brain 121, 1817–1818. 10.1093/brain/121.10.1817, PMID: 9798739

[ref89] Zatorre R. Chen J. Penhune V. (2007). When the brain plays music: auditory-motor interactions in music perception and production. Nat. Rev. Neurosci. 8, 547–558. 10.1038/nrn2152, PMID: 17585307

[ref90] Zhao T. Lam H. M. Sohi H. Kuhl P. (2017). Neural processing of musical meter in musicians and non-musicians. Neuropsychologia 106, 289–297. 10.1016/j.neuropsychologia.2017.10.007, PMID: 28987905

[ref91] Zuk J. Benjamin C. Kenyon A. Gaab N. (2014). Behavioral and neural correlates of executive functioning in musicians and non-musicians. PLoS One 9:e99868. 10.1371/journal.pone.0099868, PMID: 24937544 PMC4061064

